# Diverging Trends in Treated In-Hospital Cardiac Arrest in Intensive Care Units and General Wards Using Location-Specific Patient-Days

**DOI:** 10.3390/jcm15207881

**Published:** 2026-10-13

**Authors:** Shinhwan Lee, Kyung Hun Nam, Hang A. Park, Hye Ji Park, Yoo Ri Roh, Choung Ah Lee

**Affiliations:** 1 Department of Emergency Medicine, Hallym University Dongtan Sacred Heart Hospital, Hwaseong 18450, Republic of Korea; shinhwan.justin@gmail.com (S.L.); hangapark@gmail.com (H.A.P.); ji4051@hanmail.net (H.J.P.); 2 Department of Critical Care Medicine, Hallym University Dongtan Sacred Heart Hospital, Hwaseong 18450, Republic of Korea; nambakk@hotmail.com; 3 Clinical Quality Improvement Team, Hallym University Dongtan Sacred Heart Hospital, Hwaseong 18450, Republic of Korea; 170402@hallym.or.kr

**Keywords:** in-hospital cardiac arrest, treated cardiac arrest, incidence rate, intensive care units, general wards, patient-days

## Abstract

**Background/Objectives:** Hospital-level incidence estimates may obscure differences between intensive care units (ICUs) and general wards. We compared temporal trends in treated adult in-hospital cardiac arrest (IHCA) using location-specific patient-days. **Methods:** This retrospective study covered January 2016 to June 2026 at a South Korean teaching hospital. Monthly treated IHCA counts were modeled using negative binomial regression with patient-days as an offset, a setting × time interaction, and adjustment for the COVID-19 period and seasonality. Calendar-month score-based HAC analyses assessed robustness to serial correlation. January–March 2022 was excluded because arrest location was unavailable. **Results:** The incidence cohort comprised 1712 events: 1373 in ICUs and 339 in general wards. The combined rate was 1.06 per 1000 patient-days. The setting × time interaction was significant (monthly IRR 0.9843, model-based 95% CI 0.9768–0.9918), corresponding to a 17.3% annual decrease in the ICU-to-ward rate ratio. Calendar-month score-based HAC confidence intervals also excluded 1. ICU incidence declined (monthly IRR 0.9875, 95% CI 0.9825–0.9925), whereas the general-ward model showed no statistically significant temporal trend (IRR 1.0033, 95% CI 0.9975–1.0090; *p* = 0.263). **Conclusions:** Location-specific patient-day denominators identified different temporal trajectories in treated IHCA. These findings describe observed treated events and do not establish changes in underlying arrest risk or resuscitation performance.

## 1. Introduction

In-hospital cardiac arrest (IHCA) remains common and highly lethal, with survival-to-discharge rates of 15–25% reported across national registries [1,2,3,4]. Because a proportion of these events is preceded by recognizable physiological deterioration, IHCA incidence is increasingly used as an indicator of the quality of in-hospital care, rather than simply a measure of event burden [5,6].

Most IHCA incidence estimates use hospital admissions as the denominator. Admission-based denominators do not account for differences in length of stay. A patient hospitalized for three weeks and one admitted overnight contribute equally despite different durations of exposure. Separately, hospital-level aggregation combines settings with different baseline risks, monitoring practices, and patient populations, potentially obscuring setting-specific temporal trends.

The clinical relevance of the arrest location is well recognized. Perman et al. reported location-specific arrest counts using hospital-wide bed-days as the denominator [7]. Danish national data have reported IHCA incidence per 1000 inpatient-days as well as per admission [8], and contemporary registry analyses continue to show that arrest location is strongly associated with both incidence and outcomes [9,10]. The use of longitudinal ward-specific census data from a single institution to assess the temporal evolution of incidence in each setting over an extended period remains uncommon.

We therefore examined temporal trends in treated adult IHCA from January 2016 to June 2026 using the daily ward-specific inpatient census, complementing admission-based estimates from South Korea [11,12]. The primary question was whether temporal trends differed between ICUs and general wards. ICU outcomes and deviations during the physician workforce disruption were examined descriptively or as exploratory analyses.

## 2. Materials and Methods

### 2.1. Design and Setting

This retrospective observational study was conducted at Hallym University Dongtan Sacred Heart Hospital, a secondary-care teaching hospital in Hwaseong, Republic of Korea, which includes general wards and medical, surgical, cardiac, neurological, hybrid, and stroke intensive care units. The institutional CPR committee reviews the resuscitation events captured by the reporting system quarterly as part of a continuous quality improvement program. The study period extended from 1 January 2016 to 30 June 2026. The study reporting adhered to the STROBE statement [13].

### 2.2. Case Definition and Participants

The unit of analysis was a treated cardiac arrest event: an episode of cessation of circulation in a hospitalized patient aged 18 years or older for which chest compressions and/or defibrillation were delivered and a resuscitation report was completed, consistent with the Utstein definition for in-hospital resuscitation [14,15]. Arrests for which resuscitation was not attempted were not captured by these reports. Our estimates therefore describe treated events rather than all cardiac arrests. Arrest setting was assigned using the location field recorded in the institutional resuscitation report.

Records classified as emergency-department events were excluded from the incidence analyses. Recorded events in locations without a corresponding inpatient census, such as operating theaters, recovery areas, and procedural or imaging suites, were excluded from the incidence analyses (Supplementary Table S1). All qualifying events, including recurrent arrests in the same patient, were analyzed. Rates were expressed as events per patient-day. In a sensitivity analysis, we retained the earliest recorded treated adult arrest for each patient during the study period, identified before applying the location and quarter exclusions. Patients whose earliest event had no usable location or that occurred in the excluded quarter were omitted from this sensitivity cohort.

### 2.3. Data Sources and Consolidation

Resuscitation records were stored as quarterly spreadsheets whose data structure changed several times during the study period. Records were consolidated into a single dataset using a scripted pipeline that mapped source column headers to a standardized schema through an alias dictionary, parsed event times, and calculated intervals from recorded clock times rather than from precalculated fields. Duplicate records were removed based on the patient identifier, arrest date, and time of CPR initiation. Events for which the recorded arrest dates fell outside the corresponding reporting quarter of the source file were cross-checked against the original records and reassigned or excluded accordingly.

One quarter (January–March 2022) was extracted from a billing-code-based source that did not record arrest location. The 43 events from that quarter and the corresponding patient-days were excluded from the incidence analyses to align numerator and denominator. An additional sensitivity analysis retained those patient-days without adding location-assigned events from that quarter. This was a denominator-only scenario rather than an analysis of observed zero events.

Outcome values that were logically inferable from other records were flagged separately from observed values. Return of spontaneous circulation (ROSC) was inferred only when a documented subsequent arrest represented a distinct episode after recovery of circulation or when recorded survival information necessarily implied ROSC. Year-specific recorded counts for all observed and logically inferred adult events and missing values are presented in Supplementary Table S2. Outcome percentages used records with a known value as the denominator. Outcome data were finalized at least 30 days after the last recorded cardiac arrest, ensuring a full 30-day follow-up window for all included events.

Patient-days were calculated by summing the daily midnight census of inpatients aged 18 years or older within each included setting. This measure represents occupied census days rather than available bed-days or exact person-time. The same administrative definition was used throughout the study period. The ICU census included patient-days for a hybrid unit from 2021, a second medical ICU from 2023, and a stroke unit from 2024. Annual ICU patient-days by unit are presented in Supplementary Table S3. The wards were categorized as ICUs or general wards. To assess sensitivity to changes in unit composition, we repeated the ICU trend analysis in the medical, neurological, and surgical ICUs with documented occupancy across the study period.

### 2.4. Statistical Analysis

The primary analysis used a log-link negative binomial model for monthly event counts with log patient-days as an offset. The linear predictor included setting, calendar time in months, setting × time, a COVID-19 indicator for February 2020–December 2022, and annual sine and cosine terms. Calendar time was measured from January 2016, with its original spacing retained across the excluded quarter. General wards were the reference setting. The setting × time coefficient estimated the monthly change in the ICU-to-ward incidence rate ratio. Separate models estimated trends within each setting. Exploratory segmented models evaluated level and slope changes at the onset of the physician workforce disruption in March 2024 and the beginning of the recovery period in October 2025 following established methodological recommendations [16,17]. Setting-specific regression models were then fitted separately.

The negative binomial variance function was Var(Y) = μ + αμ^2^. In the fixed-dispersion models reported here, α was set to 1. Sensitivity analyses evaluated quarterly aggregation of counts and patient-days, a denominator-only scenario for January–March 2022, restriction to the first arrest per patient, restriction to ICU units with documented occupancy throughout the study period, and alternative nonlinear time specifications. The quarterly model used calendar-quarter time, a COVID-19 indicator for quarters containing at least one COVID-period month, and no Fourier terms. Nonlinear monthly models used quadratic time, a continuous piecewise trend with a knot at January 2018, and a restricted cubic spline with boundary knots at January 2016 and June 2026 and one interior knot at February 2021. Joint Wald tests evaluated the setting interactions with all nonconstant time terms, with 2 df for each nonlinear specification. ICU characteristics and outcomes were summarized descriptively. Outcome percentages used records with a known value as the denominator.

As an additional analysis, we calculated a calendar-month score-based heteroscedasticity- and autocorrelation-consistent covariance estimator. Scores for the ICU and general-ward observations were summed within each month. Bartlett weights with maximum lags of 6 and 12 months were applied using the original calendar spacing, including the gap for January–March 2022. Normal Wald intervals were calculated without a finite-sample multiplier. The sandwich bread was the inverse observed information of the fixed-α negative binomial mean model.

Annual incidence rates were accompanied by exact Poisson confidence intervals. Descriptive ICU-to-ward rate ratios used exact conditional intervals. These intervals assume independent Poisson event counts and do not account for recurrent events or overdispersion. Annual changes in model-based rates were calculated as 100 × [exp(12β) − 1] using unrounded monthly coefficients.

The analyses were performed using Python 3.12.3 (Python Software Foundation, Beaverton, OR, USA) with the open-source statsmodels package (version 0.15.0). Statistical significance was defined as *p* < 0.05.

### 2.5. Ethics

This study was conducted in accordance with the principles of the Declaration of Helsinki and approved by the Institutional Review Board of Hallym University Dongtan Sacred Heart Hospital (IRB 2026-01-007) on 2 February 2026. The requirement for written informed consent was waived by the IRB because of the retrospective study design and use of routinely collected data.

## 3. Results

### 3.1. Events and Denominators

A total of 1877 treated adult resuscitation events were recorded during the 126-month study period (Supplementary Table S4). The incidence cohort included 1712 events: 1373 in ICUs and 339 in general wards. The remaining 165 events were excluded from incidence analyses, comprising 43 events from January–March 2022 with unavailable location information and 122 other events with unusable locations or without aligned location-specific denominators.

The analyzed periods comprised 176,433 ICU patient-days and 1,445,003 general-ward patient-days. The combined incidence rate for these two settings was 1.06 treated events per 1000 patient-days.

### 3.2. Incidence by Setting

**Figure 1:**
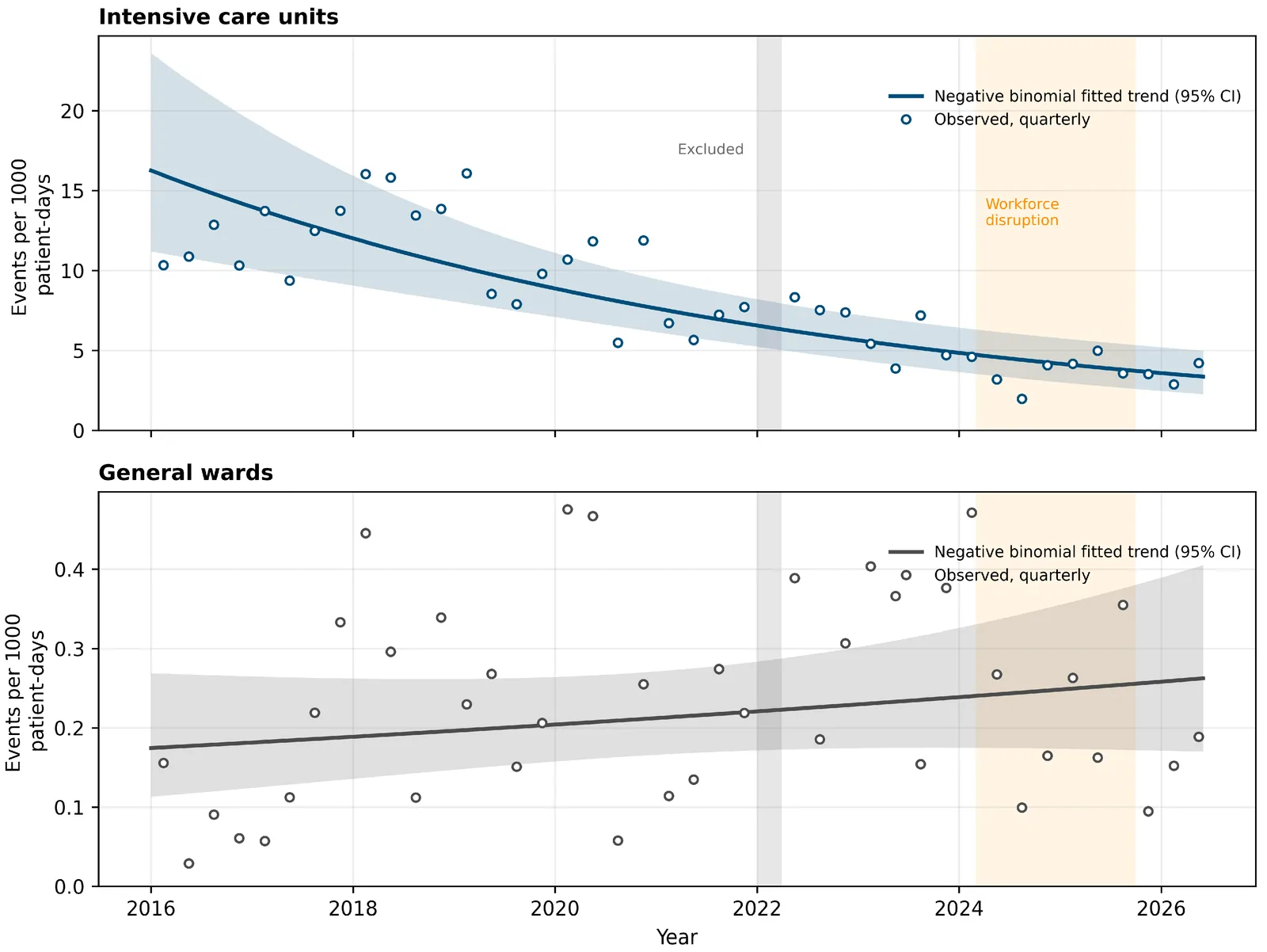
Quarterly incidence of treated adult IHCA in ICUs and general wards, January 2016 to June 2026. Note: Rates are treated events per 1000 setting-specific patient-days. The upper panel shows ICUs and the lower panel general wards, with different y-axis scales. Points show observed quarterly rates. Lines and pointwise 95% confidence bands were derived from separate ICU and ward monthly negative binomial models using model-based covariance based on the inverse expected Fisher information. For prediction, the COVID-19 indicator and both annual Fourier terms were set to zero. The bands describe uncertainty in the fitted mean rates and differ from confidence intervals for regression coefficients, including the setting × time interaction. January–March 2022 was excluded and is marked by the gray band. Fitted values over that interval are model predictions, not observed rates. The orange band marks the physician workforce disruption from March 2024 to September 2025 for context.

Treated IHCA incidence was higher in ICUs than in general wards (Table 1, Figure 1). The ICU rate was highest in 2018, at 14.78 events per 1000 ICU patient-days, and was 3.53 in January–June 2026. General-ward rates ranged from 0.083 to 0.324 events per 1000 general-ward patient-days. The descriptive ICU-to-general-ward rate ratio was 133.3 (95% CI 72.1–273.4) in 2016 and 20.7 (10.5–44.7) in January–June 2026. Each endpoint included only 11 general-ward events, the 2016 ratio was the highest in the series, and the 2026 estimate covered six months. The setting × time interaction provides the primary model-based assessment of divergence.

### 3.3. Divergence of Trends

The setting × time interaction indicated different temporal trends between ICUs and general wards (monthly IRR 0.9843, model-based 95% CI 0.9768–0.9918; *p* < 0.001). This corresponds to a 17.3% annual decrease in the ICU-to-general-ward incidence rate ratio calculated from the unrounded coefficient (Table 2).

In separate setting-specific models, ICU treated IHCA incidence declined by 14.1% annually (monthly IRR 0.9875, 95% CI 0.9825–0.9925; *p* < 0.001). The general-ward model showed no statistically significant temporal trend (IRR 1.0033, 95% CI 0.9975–1.0090; *p* = 0.263). With restriction to the medical, neurological, and surgical ICUs, 941 events and 132,591 patient-days were included across 123 months. The monthly ICU trend IRR was 0.9874 (95% CI 0.9822–0.9926; *p* < 0.001) (Table 2). This restriction addresses changes in unit composition, but does not remove possible changes in case mix, severity, admission criteria, or resuscitation-status decisions within those units.

Calendar-month score-based HAC intervals for the interaction also excluded 1: 0.9793–0.9894 with a 6-month lag and 0.9789–0.9898 with a 12-month lag. The point estimate remained 0.9843 because the fitted mean model was unchanged. In the first-arrest-per-patient sensitivity cohort, 1267 events were retained (953 in ICUs and 314 in general wards). The interaction IRR was 0.9862 (95% CI 0.9786–0.9939; *p* < 0.001) (Table 2).

Sensitivity analyses evaluated quadratic time, a piecewise trend with a knot at January 2018, and a restricted cubic spline with one interior knot at February 2021. Joint Wald tests of the setting interactions with the nonconstant time terms supported different temporal trajectories under each specification (*p* = 0.000174, 0.000106, and 0.000188, respectively) (Table 2). The denominator-only scenario yielded an interaction IRR of 0.9844 (95% CI 0.9769–0.9919), and quarterly aggregation yielded an IRR of 0.9535 per quarter (95% CI 0.9191–0.9893) (Table 2).

### 3.4. Aggregate versus Location-Specific Incidence Trends

Combining ICU and general-ward events yielded a single estimated decline whether hospital admissions or combined patient-days were used as the denominator (Table 3). Separate setting-specific models identified an ICU decline and no statistically significant general-ward trend, indicating that aggregation can obscure differences between settings.

To illustrate denominator scope, the same included counts were also divided by the combined ICU and general-ward patient-days (Supplementary Table S5). This yielded 0.847 ICU events and 0.209 general-ward events per 1000 combined patient-days compared with 7.782 and 0.235 using setting-specific denominators. These descriptive calculations do not establish comparability with an external registry.

### 3.5. Exploratory Analyses

Exploratory models examined level and slope changes at the onset of the physician workforce disruption in March 2024 and the beginning of the recovery period in October 2025. Detailed estimates are provided in Supplementary Table S6. All eight disruption- and recovery-related level and slope change estimates had 95% confidence intervals that included 1, and several intervals were wide. These results are imprecise and do not establish an absence of effects.

Characteristics and outcomes of treated ICU arrests are summarized descriptively in Table 4. Outcome proportions varied across years, with higher values in January–June 2026 than in 2017. The estimates were unadjusted for initial rhythm, comorbidity, illness severity, and changes in patient selection. Missing outcome data, particularly for 30-day survival in 2019, also limit comparison. These observations should not be interpreted as evidence of improved resuscitation performance. Outcome-field completeness for this ICU cohort is presented in Supplementary Table S2, Panel B.

## 4. Discussion

Over ten and a half years at a single institution, ICU treated IHCA incidence declined by 14.1% annually in the setting-specific model, whereas the general-ward model showed no statistically significant temporal trend. The setting × time interaction indicated different trajectories. Combining the two settings yielded a single estimated decline and obscured this difference.

Changes in critical-care practice and monitoring [18,19], patient case mix, ICU admission thresholds, and decisions to attempt resuscitation could have contributed to the observed decline. Our data cannot distinguish these mechanisms. Earlier treatment limitation could have reduced the number of treated events without reducing the underlying incidence of circulatory arrest. South Korean life-sustaining treatment legislation provides relevant context [20], but our dataset cannot quantify its contribution at this institution.

The general-ward pattern warrants further investigation, but it does not identify a failure of deterioration recognition or escalation. Rapid-response systems provide relevant context for future evaluation [21,22,23,24], but we did not measure their activation, monitoring practices, or time to escalation. Early-warning score performance and automated detection could also be examined in future studies [25,26], together with patient case mix and resuscitation-status decisions.

Denominator selection and the level of aggregation address different questions. Patient-days account for exposure duration, while separation by clinical setting allows distinct trajectories to be assessed. In our data, aggregate rates declined with either denominator, but setting-specific analysis identified an ICU decline and no statistically significant general-ward trend. Linking census data with resuscitation reports may therefore improve the description of institutional event burden.

Our absolute rates are not directly comparable with those of Perman et al. [7], who divided location-specific event counts by hospital-wide bed-days. We used patient-days within each setting. Applying our combined ICU and general-ward denominator to the same counts yields 0.847 ICU events and 0.209 general-ward events per 1000 patient-days (Supplementary Table S5). This illustrates the arithmetic effect of denominator scope rather than a standardized cross-study comparison.

Patient populations, unit definitions, study period, and aggregation of hospital rates also limit comparability. Potential differences in event-counting rules or ascertainment were not established and require reconciliation before direct comparison. We cannot quantify their contribution to the remaining difference. Consistent institutional definitions support comparison over time, but changes in case mix, DNAR practices, documentation, or unit composition could still have influenced temporal trends.

The physician workforce disruption provides national context for the exploratory analysis [27]. Estimates from a single institution reflect its admitted population and cannot establish national effects. The workforce estimates are reported separately in Supplementary Table S6 and should be interpreted with their uncertainty.

### Limitations

This study had several methodological limitations. It was conducted at a single hospital, and the trends should not be extrapolated to national IHCA incidence. Our case definition captured treated arrests only. The dataset does not identify all arrests for which CPR was withheld. Without longitudinal pre-arrest resuscitation-status information, we cannot quantify changes in DNAR practices or their contribution to the observed trends.

Emergency-department events were outside the incidence cohort, limiting the coverage of hospital-wide treated arrests. Recorded events in operating theaters, recovery areas, and procedure or imaging suites were excluded from incidence analyses because aligned census denominators were unavailable. January–March 2022 was excluded because location information was missing.

The initial cardiac arrest rhythm was not recorded before 2023 and remained incompletely recorded thereafter. We could not adjust for arrest rhythm or assess changes in its distribution. Patient-level illness severity and comorbidity data were unavailable, so changes in case mix within each setting could not be quantified. ICU outcomes were unadjusted descriptive results, and variation in missingness limits comparisons across years. Patient-days were derived from a consistently recorded midnight census rather than exact time at risk. Same-day admissions and discharges and within-day transfers may be incompletely represented, and changes in bed turnover could alter this measurement error over time or between settings. We could not quantify its magnitude or temporal variation from the available data.

## 5. Conclusions

At a single South Korean teaching hospital, treated IHCA incidence declined in ICUs, whereas no statistically significant temporal trend was identified in general wards. The setting × time interaction indicated different trajectories. Location-specific patient-day denominators revealed differences obscured by aggregation. The causes of these trends and their relation to underlying arrest risk or care quality require further study.

## Figures and Tables

**Table 1:** Annual incidence rate of treated adult in-hospital cardiac arrest per 1000 inpatient-days by setting.

Year	Ward Patient-Days	Ward Events	Ward Rate (95% CI)	ICU Patient-Days	ICU Events	ICU Rate (95% CI)	Ratio (95%CI)
2016	132,636	11	0.083 (0.041–0.148)	12,122	134	11.05 (9.26–13.09)	133.3 (72.1–273.4)
2017	142,935	26	0.182 (0.119–0.267)	12,685	156	12.30 (10.44–14.39)	67.6 (44.4–106.7)
2018	143,695	43	0.299 (0.217–0.403)	14,615	216	14.78 (12.87–16.89)	49.4 (35.5–70.2)
2019	158,619	34	0.214 (0.148–0.300)	14,757	156	10.57 (8.98–12.37)	49.3 (33.9–73.7)
2020	139,802	44	0.315 (0.229–0.423)	16,664	166	9.96 (8.50–11.60)	31.7 (22.6–45.2)
2021	145,063	27	0.186 (0.123–0.271)	18,640	128	6.87 (5.73–8.16)	36.9 (24.2–58.1)
2022 ‡	115,401	34	0.295 (0.204–0.412)	15,113	117	7.74 (6.40–9.28)	26.3 (17.8–39.7)
2023	154,163	50	0.324 (0.241–0.428)	21,445	114	5.32 (4.38–6.39)	16.4 (11.7–23.3)
2024	124,285	32	0.257 (0.176–0.363)	20,211	71	3.51 (2.74–4.43)	13.6 (8.9–21.4)
2025	123,771	27	0.218 (0.144–0.317)	18,550	74	3.99 (3.13–5.01)	18.3 (11.6–29.6)
2026 H1	64,633	11	0.170 (0.085–0.305)	11,631	41	3.53 (2.53–4.78)	20.7 (10.5–44.7)
Total	1,445,003	339	0.235 (0.210–0.261)	176,433	1373	7.78 (7.38–8.20)	33.2 (29.4–37.5)

Rates are treated events per 1000 setting-specific patient-days. Rate ratio = ICU rate ÷ general-ward rate. Rate intervals are exact Poisson intervals and ratio intervals are exact conditional intervals under independent Poisson counts. These are descriptive and do not adjust for recurrent arrests or overdispersion. ‡ January–March 2022 was excluded from both counts and patient-days. H1 denotes January–June.

**Table 2:** Temporal trends in treated IHCA and sensitivity analyses.

Analysis	IRR (95% CI)	*p*
Setting × time interaction, model-based	0.9843 (0.9768–0.9918)	<0.001
Interaction, calendar-month HAC lag 6	0.9843 (0.9793–0.9894)	<0.001
Interaction, calendar-month HAC lag 12	0.9843 (0.9789–0.9898)	<0.001
Separate ICU model, model-based	0.9875 (0.9825–0.9925)	<0.001
Separate general-ward model, model-based	1.0033 (0.9975–1.0090)	0.263
First arrest per patient, interaction	0.9862 (0.9786–0.9939)	<0.001
2022 Q1 denominator-only, interaction	0.9844 (0.9769–0.9919)	<0.001
Quarterly aggregation, interaction §	0.9535 (0.9191–0.9893)	0.011
MICU, NCU and SICU, ICU trend	0.9874 (0.9822–0.9926)	<0.001
Quadratic time †	—	0.000174
Piecewise time, knot at January 2018 †	—	0.000106
Restricted cubic spline, 2 time df †	—	0.000188

IRRs are per month unless otherwise stated. Models used the NB2 variance function with α fixed at 1 and log patient-days as the offset. Monthly models included a COVID-19 indicator for February 2020–December 2022 and annual sine and cosine terms. Model-based intervals used the inverse expected Fisher information. HAC intervals used inverse observed-information bread, within-month summed scores, Bartlett weights, and calendar-month lags of 6 or 12, preserving the excluded-quarter gap. § The quarterly model included a COVID-19 indicator equal to 1 when a quarter contained at least one COVID-period month and did not include Fourier terms. Its interaction IRR is per quarter. The equivalent monthly IRR is 0.9843 (95% CI 0.9723–0.9964), calculated by dividing the coefficient and confidence limits on the log scale by 3. † Joint Wald tests assessed the setting interactions with the nonconstant time terms: 2 df for quadratic, piecewise, and restricted cubic spline specifications. The spline used boundary knots at January 2016 and June 2026 and one interior knot at February 2021. A single IRR is not interpretable for these nonlinear specifications.

**Table 3:** Effect of denominator scope and aggregation on estimated temporal trends.

Denominator	Event Population	Monthly IRR (95% CI)	Annual Change
Hospital admissions	Combined ICU and general ward	0.9926 (0.9877–0.9976)	−8.5%
Combined patient-days	Combined ICU and general ward	0.9944 (0.9895–0.9994)	−6.5%
ICU patient-days	ICU only	0.9875 (0.9825–0.9925)	−14.1%
General-ward patient-days	General ward only	1.0033 (0.9975–1.0090)	+4.0%

Models used NB2 α fixed at 1, the COVID-19 indicator, and annual Fourier terms. The two aggregate models used the same combined ICU and general-ward event cohort and excluded January–March 2022. Hospital admissions were counted once per calendar month. Setting-specific models used the corresponding setting counts and patient-days. Annual changes were calculated from unrounded coefficients as 100 × [exp(12β) − 1].

**Table 4:** Characteristics and outcomes of treated ICU cardiac arrests by year.

Year	Events	ROSC *n*/N (%)	24 h Alive *n*/N (%)	30 d Alive *n*/N (%)	CPR min Median (*n*)	Age Years Mean (*n*)
2017	156	92/156 (59.0%)	38/156 (24.4%)	20/156 (12.8%)	9.5 (*n* = 156)	67.13 (*n* = 156)
2018	216	108/216 (50.0%)	49/216 (22.7%)	24/215 (11.2%)	13.0 (*n* = 216)	67.16 (*n* = 216)
2019	156	93/153 (60.8%)	34/153 (22.2%)	12/115 (10.4%)	9.0 (*n* = 155)	66.36 (*n* = 156)
2020	166	102/166 (61.4%)	40/165 (24.2%)	15/165 (9.1%)	10.0 (*n* = 166)	67.49 (*n* = 166)
2021	128	82/125 (65.6%)	36/123 (29.3%)	18/123 (14.6%)	12.0 (*n* = 127)	68.81 (*n* = 128)
2022 ‡	117	91/117 (77.8%)	42/117 (35.9%)	20/117 (17.1%)	10.0 (*n* = 117)	68.12 (*n* = 117)
2023	114	74/108 (68.5%)	34/108 (31.5%)	15/107 (14.0%)	11.0 (*n* = 114)	69.52 (*n* = 114)
2024	71	45/69 (65.2%)	21/69 (30.4%)	8/66 (12.1%)	15.0 (*n* = 71)	67.35 (*n* = 71)
2025	74	48/74 (64.9%)	26/74 (35.1%)	13/73 (17.8%)	15.5 (*n* = 74)	71.28 (*n* = 74)
2026 H1	41	34/41 (82.9%)	18/41 (43.9%)	9/41 (22.0%)	7.0 (*n* = 41)	71.49 (*n* = 41)

Outcome percentages are *n*/N among records with a known outcome; N may include logically inferred values and differs by outcome. Missing outcomes were not counted as failures. Completeness for this ICU cohort is shown in Supplementary Table S2, Panel B. CPR duration is the median among records with available duration, and age is the mean among records with available age. The 2016 row was omitted from this cross-outcome comparison because ROSC was not directly recorded and 30-day survival was unavailable. Recorded 24 h survival in 2016 was 28/134 (20.9%). The 58 inferred ROSC-positive records do not provide an unbiased estimate of the 2016 ROSC proportion. ‡ January–March 2022 was excluded. H1 denotes January–June. Data for 2026 cover January–June and include 41 events, the smallest annual sample in the table. Each event had an opportunity for at least 30 days of follow-up. Outcome estimates should be interpreted in view of the small sample and the partial-year event-accrual period. CPR duration was missing for 2 ICU events.

## Data Availability

The raw data supporting the conclusions of this article will be made available by the authors on request.

## Supplementary Materials

The following supporting information can be downloaded at https://www.mdpi.com/article/10.3390/jcm15207881/s1. Table S1: Classification of arrest locations; Table S2: Completeness of outcome fields by year; Table S3: Intensive care unit patient-days by unit and year; Table S4: Classification of resuscitation events; Table S5: Effect of denominator scope on descriptive treated IHCA rates; Table S6: Exploratory segmented analyses of physician workforce disruption.

## Abbreviations

IHCA	in-hospital cardiac arrest
ICU	intensive care unit
IRR	incidence rate ratio
ROSC	return of spontaneous circulation
CPR	cardiopulmonary resuscitation
DNAR	do-not-attempt-resuscitation
