# Supplementary material for: Diverging Trends in Treated In-Hospital Cardiac Arrest in Intensive Care Units and General Wards Using Location-Specific Patient-Days
Source: J Clin Med. 2026 Oct 13;15(20):7881. doi: 10.3390/jcm15207881 (PMC13705293; doi:10.3390/jcm15207881)
Supplement: Supplementary file 1 [file jcm-15-07881-s001.zip › jcm-4577031-supplementary.pdf]

Supplementary Materials

Diverging Trends in Treated In-Hospital Cardiac Arrest in Intensive Care Units and General Wards Using Location-Specific Patient-Days

Contents

Table S1. Classification of arrest locations

Table S2. Completeness of outcome fields by year: Panel A, all recorded adult events; Panel B, incidence-cohort ICU events

Table S3. Intensive care unit patient-days by unit and year

Table S4. Classification of resuscitation events

Table S5. Effect of denominator scope on descriptive treated IHCA rates

Table S6. Exploratory segmented analyses of physician workforce disruption

Table S1. Classification of arrest locations

Classification	Location categories	Events
Intensive care units	Medical, surgical, neurological, cardiac, hybrid and stroke ICUs, including ICU events without a specified unit	1,373
General wards	Adult general wards identified by ward names or numbered ward codes	339
Other or unusable locations	Emergency department, operating/recovery areas, procedural or imaging areas, dialysis units, outpatient/treatment areas, corridors, or missing locations	165

Source aliases were mapped to institutional census codes, with case variants treated as equivalent. ICU events without an assignable unit were retained in the overall ICU cohort but excluded from the MICU–NCU–SICU sensitivity cohort. The 47 events with missing location information were excluded: 43 from January–March 2022 and four from other periods.

Table S2. Completeness of outcome fields by year

Panel A. All recorded adult resuscitation events

Year	Events	ROSC			24-hour survival			30-day survival		
		Obs.	Inf.	Miss.	Obs.	Inf.	Miss.	Obs.	Inf.	Miss.
2016	159	0	74	85	159	0	0	0	0	159
2017	196	196	0	0	196	0	0	195	0	1
2018	265	265	0	0	265	0	0	263	0	2
2019	202	199	0	3	199	0	3	147	0	55
2020	221	220	1	0	220	0	1	220	0	1
2021	172	166	3	3	166	0	6	166	0	6
2022	204	203	0	1	203	0	1	203	0	1
2023	172	165	0	7	165	0	7	164	0	8
2024	116	113	1	2	113	0	3	108	0	8
2025	112	112	0	0	112	0	0	111	0	1
2026 (H1)	58	58	0	0	58	0	0	58	0	0
Total	1,877	1,697	79	101	1,856	0	21	1,635	0	242

Panel B. ICU events included in the incidence cohort

Year	Events	ROSC			24-hour survival			30-day survival		
		Obs.	Inf.	Miss.	Obs.	Inf.	Miss.	Obs.	Inf.	Miss.
2016	134	0	58	76	134	0	0	0	0	134
2017	156	156	0	0	156	0	0	156	0	0
2018	216	216	0	0	216	0	0	215	0	1
2019	156	153	0	3	153	0	3	115	0	41
2020	166	165	1	0	165	0	1	165	0	1
2021	128	123	2	3	123	0	5	123	0	5
2022‡	117	117	0	0	117	0	0	117	0	0
2023	114	108	0	6	108	0	6	107	0	7
2024	71	69	0	2	69	0	2	66	0	5
2025	74	74	0	0	74	0	0	73	0	1
2026 (H1)	41	41	0	0	41	0	0	41	0	0

Obs., recorded in the source file; Inf., not recorded but logically inferred; Miss., not available. Panel A includes all recorded adult events. Panel B includes the ICU incidence cohort, excluding January–March 2022 (‡). Data for 2026 cover January–June.

Logically inferred values were flagged separately from recorded values. ROSC was inferred only when a documented subsequent arrest represented a distinct episode after recovery of circulation, or when recorded survival information necessarily implied ROSC. Outcome percentages used known records as the denominator; missing outcomes were not counted as failures.

In the ICU cohort in 2016, ROSC was not directly recorded: 58 values were inferred positive and 76 were missing. Recorded 24-hour survival was available for all 134 ICU events, whereas 30-day survival was unavailable. The inferred ROSC-positive subset does not provide an unbiased estimate of that year's ROSC proportion. Outcome variables were not used in the primary incidence model.

Table S3. Intensive care unit patient-days by unit and year

Year	CCU	HICU	MICU	MICU2	NCU	SICU	STU	Total ICU
2016	0	0	5,008	0	2,378	4,736	0	12,122
2017	572	0	4,866	0	2,846	4,401	0	12,685
2018	2,595	0	4,486	0	2,713	4,821	0	14,615
2019	2,646	0	4,841	0	2,754	4,516	0	14,757
2020	2,906	0	4,963	0	3,870	4,925	0	16,664
2021	2,827	1,390	4,989	0	4,653	4,781	0	18,640
2022	3,011	1,392	5,186	0	5,232	5,208	0	20,029
2023	2,886	1,339	5,001	2,110	4,994	5,115	0	21,445
2024	2,793	1,322	3,799	3,332	3,956	4,407	602	20,211
2025	2,513	1,366	3,290	3,034	3,278	3,789	1,280	18,550
2026 (H1)	1,419	830	2,089	1,895	2,171	2,357	870	11,631

Values are occupied census-days for patients aged 18 years or older. CCU, cardiac care unit; HICU, hybrid intensive care unit; MICU, medical intensive care unit; MICU2, second medical intensive care unit; NCU, neurological care unit; SICU, surgical intensive care unit; STU, stroke unit. Table S3 presents annual administrative census totals without applying the primary analysis quarter exclusion. The primary incidence analyses excluded January–March 2022, leaving 15,113 ICU patient-days for 2022 compared with 20,029 in this table. Data for 2026 cover January–June.

Patient-days were recorded for a hybrid unit from 2021, a second medical unit from 2023, and a stroke unit from 2024. Total ICU patient-days increased from 12,122 in 2016 to 21,445 in 2023, a 76.9% increase. Sensitivity to unit composition was assessed by restricting the ICU analysis to the medical, neurological, and surgical ICUs with documented occupancy across the study period. This restriction cannot eliminate changes in patient case mix, severity, admission thresholds, or resuscitation-status decisions within those units.

Table S4. Classification of resuscitation events

Panel A. Classification of all recorded events

Step	n
Treated adult resuscitation events, January 2016–June 2026	1,877
Unique patients in all recorded adult events	1,416
Additional events beyond the first per patient in all recorded events	461
Assigned to an intensive care unit	1,373
Assigned to a general ward	339
Excluded from incidence analyses	165
Other events with unusable locations or without aligned location-specific denominators	122
January–March 2022: location not recorded	43
Included in incidence analyses	1,712

Panel B. First-event sensitivity flow

Step	n
First event selected per patient before exclusions	1,416
Excluded for January–March 2022	38
Other unusable or unaligned locations	111
First events included in incidence analyses	1,267
ICU first events	953
General-ward first events	314

Incidence analyses included every eligible treated event, including additional arrests in the same patient, because rates were expressed per patient-day. Counts of unique patients and additional events in this table refer to all recorded adult events and are not the size of the first-event incidence sensitivity cohort. Events with unusable locations or without aligned location-specific denominators were excluded. For January–March 2022, the billing-code extract contained no location field; those events and the corresponding patient-days were removed from both numerator and denominator. The first-event sensitivity cohort was selected according to the rule specified in the main-text Methods. Of 1,416 selected first events, 38 were excluded for January–March 2022 and 111 for other unusable or unaligned locations, leaving 1,267 events (953 in ICUs and 314 in general wards).

Table S5. Effect of denominator scope on descriptive treated IHCA rates

Events	Denominator scope	ICU rate	Ward rate
All included events	Setting-specific patient-days	7.782	0.235
All included events	Combined ICU and ward patient-days	0.847	0.209

Rates are treated events per 1,000 patient-days. Both rows use the same included counts: 1,373 ICU events and 339 general-ward events. Setting-specific denominators are 176,433 ICU patient-days and 1,445,003 general-ward patient-days; the combined denominator is 1,621,436 patient-days. The combined denominator is used to illustrate denominator scope and does not establish equivalence with an external registry denominator. January–March 2022 was excluded from counts and patient-days.

Table S6. Exploratory segmented analyses of physician workforce disruption

Setting and term	IRR (95% CI)	p
ICU — Level change at onset	0.556 (0.185–1.669)	0.295
ICU — Monthly slope change at onset	1.032 (0.944–1.129)	0.489
ICU — Level change at recovery	0.672 (0.124–3.627)	0.644
ICU — Monthly slope change at recovery	1.011 (0.751–1.362)	0.941
General ward — Level change at onset	0.508 (0.150–1.716)	0.275
General ward — Monthly slope change at onset	1.011 (0.915–1.117)	0.830
General ward — Level change at recovery	0.278 (0.034–2.257)	0.231
General ward — Monthly slope change at recovery	1.096 (0.757–1.587)	0.626

Separate monthly log-link NB2 models were fitted for ICUs and general wards, with α fixed at 1 and log patient-days as the offset. Each model included calendar time, the COVID-19 indicator for February 2020–December 2022, annual sine and cosine terms, and step and slope-change terms at March 2024 and October 2025. Calendar time t was measured in months from January 2016, retaining the original spacing across the excluded quarter. The onset step was $I(t \geq 98)$, the onset slope term was $\max(t - 98, 0)$, the recovery step was $I(t \geq 117)$, and the recovery slope term was $\max(t - 117, 0)$. Confidence intervals and p values used model-based covariance from the inverse expected Fisher information and normal Wald inference. Level-change IRRs describe an immediate change relative to the preceding segment extrapolated to the transition month. Slope-change IRRs describe the ratio of the monthly trend IRRs after and before each transition. The disruption period was March 2024–September 2025, and the recovery period began in October 2025. Estimates describe the admitted population at this institution and should not be interpreted as national effects.
